# Multiplexed Detection of *Salmonella*, *Escherichia coli*, *Campylobacter,* and *Listeria* in Raw Poultry

**DOI:** 10.3390/foods14071137

**Published:** 2025-03-25

**Authors:** Chin-Yi Chen, Cheryl M. Armstrong, Yiping He, Katrina Counihan, Javier Atencia, Joe Lee, Gretchen Dykes, Kathleen Johnson, Heather Koppenhofer, Shannon Tilman, Sean Martinos, Joseph Capobianco

**Affiliations:** 1 United States Department of Agriculture, Agricultural Research Service, Characterization and Interventions for Foodborne Pathogens, Wyndmoor, PA 19038, USA; chin-yi.chen@usda.gov (C.-Y.C.); cheryl.armstrong@usda.gov (C.M.A.); yiping.he@usda.gov (Y.H.); katrina.counihan@usda.gov (K.C.); joe.lee@usda.gov (J.L.); gretchen.dykes@usda.gov (G.D.); kathleen.m.johnson@usda.gov (K.J.); heather.koppenhofer@usda.gov (H.K.); shannon.tilman@usda.gov (S.T.); 2 Pathotrak Inc., Laurel, MD 20707, USA; smartinos@pathotrak.com

**Keywords:** foodborne pathogens, food safety, sample preparation, enrichment-free

## Abstract

The detection of foodborne pathogens is a critical aspect of ensuring food safety. Traditional methods rely on time-intensive enrichment steps and pathogen-specific assays, extending testing timelines and limiting throughput. This study evaluates an enrichment-free, multiplexed pathogen detection workflow combining the Pathotrak system for bacterial separation and the Neogen Molecular Detection System (MDS) for detection. The workflow enables simultaneous detection of *Salmonella*, *Escherichia coli* O157, *Listeria monocytogenes*, *Listeria* spp., and *Campylobacter* in poultry samples, significantly reducing the time taken to screen samples requiring further evaluation. The system’s performance was assessed using inoculated chicken samples over a range of bacterial concentrations (10^2^–10^8^ CFU/mL). The MDS system demonstrated robust detection for most pathogens, with strong correlations between theoretical inoculation levels and MDS-calculated concentrations (R^2^ > 0.85 for all pathogens). However, detection variability was observed at lower concentrations for *Salmonella* and *Listeria monocytogenes*. The system maintained high sensitivity and specificity, achieving a Cohen’s Kappa coefficient for *E. coli* and *Campylobacter*. This study highlights the potential of enrichment-free, multiplex detection to streamline food safety testing by reducing the time to results, enhancing efficiency, and providing reliable pathogen quantification across multiple targets.

## 1. Introduction

Food safety is a critical public health priority, as foodborne pathogens pose significant risks to consumers, leading to outbreaks, hospitalizations, and long-term health complications. Ensuring the detection and mitigation of these pathogens is essential not only for regulatory compliance and maintaining industry standards but also for protecting public health and reducing the burden of foodborne illnesses globally [1]. Because foodborne pathogens are typically present in low numbers, food safety diagnostics generally rely on methods that increase the concentration of target pathogens to improve their detection probability [2]. Enrichment is one such process, and aims to create an environment conducive to the rapid growth of specific microorganisms. It is one of the most common techniques employed to increase bacterial concentrations [3] and has many advantages considering it is cost-effective, simple to apply, and distinguishes live cells from those that were eliminated by intervention strategies [4].

Conversely, there are several drawbacks to culture enrichment as well. First, the process can be labor intensive and, therefore, not suited for high-throughput screening methodologies [5]. It is also time consuming, taking many hours and sometimes several days to complete [4]. In addition, the inherent complexity within and amongst microbial genera introduces immense challenges since the conditions needed for the successful growth of the different organisms can vary. For example, the culture conditions (i.e.,: media, temperature, and enrichment times) used by the United States Department of Agriculture’s Food Safety and Inspection Service (FSIS) are aimed at amplifying *Salmonella* from raw chicken products, and are not the same as those used for *Listeria* from ready-to-eat products [6,7]. Moreover, even under conditions intended to promote the robust growth of a species such as *Salmonella enterica*, different *Salmonella* serovars can vary in their response to the enrichment conditions due to differences in their ability to recover from stress, replicate, and compete with background microbes [8,9,10]. As a result, detection methods employing culture enrichment procedures may demonstrate biases, potentially leading to false negatives or the incomplete assessment of the microbial composition within a sample [11,12,13]. Consequently, multiple selective media may be utilized during the enrichment procedure when surveying food samples in an effort to identify all potential pathogens present [6,14]. Therefore, despite the success pathogen screening has achieved through culture enrichment, the process is less than ideal. This has led both producers and regulators to seek alternative methods that can streamline the detection process, increase process efficiency, and avoid biases [15].

Other forms of sample preparation such as isolation, concentration, and purification may also be used in conjunction with food safety diagnostics to improve the probability of detecting pathogens from within food matrices. Successful applications of these processes often incorporate sample preparation techniques for separating target microorganisms from non-target bacteria and inhibitory substances with the ability to concentrate the pathogens to levels that exceed the detection limit of downstream assays such as the polymerase chain reaction (PCR) or loop-mediated isothermal amplification (LAMP). Such procedures typically involve subsampling, and a homogeneous suspension must be produced to ensure that the act of subsampling does not introduce variability/bias. This is critical since food samples, such as a 400 mL poultry rinse collected following FSIS Directive 10,250.1, contain different types of tissues and varied microbiomes [16,17], which are not uniformly distributed through the sample volume. Concentrating samples to volumes within the microliter range can be an effective means of minimizing subsampling errors because this can allow the entire sample to be applied to a downstream molecular screening platform. Unfortunately, most methods capable of concentrating samples to within this range were not designed to accommodate complex matrices such as those inherent to food samples.

The Pathotrak Next Generation Extraction (NGE) System was developed to provide an alternative to traditional enrichment-based methods by utilizing a direct extraction approach. This method aims to reduce the potential biases associated with selective enrichment while enabling faster pathogen detection. The NGE system completes the extraction process in under three hours for up to 96 samples, significantly shortening the preparation time required for subsequent molecular analysis compared to conventional overnight enrichment methods. Additionally, the system is designed to accommodate high-throughput testing by eliminating the need for pathogen-specific enrichment media and extended incubation times. An elution from the Pathotrak’s NGE system is compatible with a broad range of downstream microbial detection platforms, including immunoassays and molecular assays, which can be multiplexed for simultaneous detection of microorganisms, including *Salmonella*, *Escherichia coli* O157, *Listeria monocytogenes*, and *Campylobacter* species. For the purposes of establishing proof of principle, this study utilized a commercial loop-mediated isothermal amplification (LAMP) assay and the Neogen Molecular Detection System (MDS), and explored an analysis of the response to provide a quantitative response for threshold determination.

## 2. Materials and Methods

**Figure 1:**
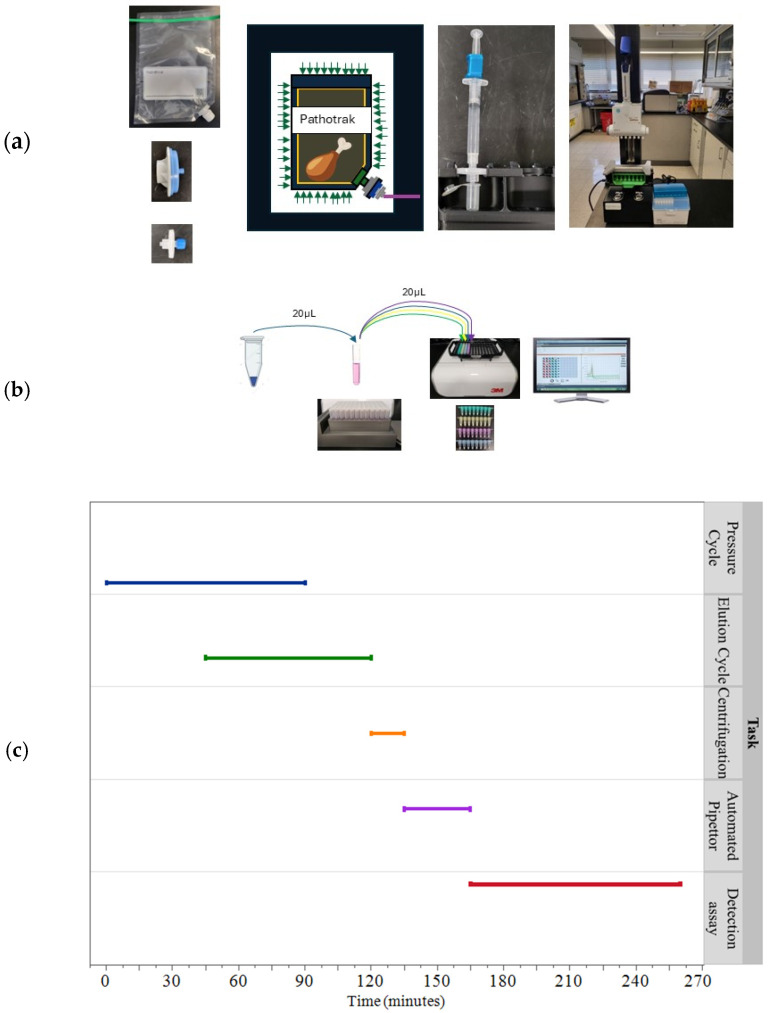
Pathotrak workflow and integration with Neogen MDS. (**a**) The figure illustrates the workflow of the NGE system, starting with the introduction of a poultry sample into a Pathotrak bag equipped with a capture membrane. The bag is subjected to isostatic pressure within a Pathotrak pod, facilitating the separation of target pathogens from the bulk sample. Bacteria captured by the membrane are subsequently eluted, and the eluate is concentrated to a target volume of 20 µL using centrifugation and an automated pipettor. (**b**) The concentrated sample is added directly to the lysis buffer of the Neogen MDA, which is then transferred to reagent tubes specific to pathogen detection. (**c**) The accompanying graph depicts the time allocation for each processing step and the multiplexed detection process for 96 samples.

Figure 1 illustrates the Pathotrak workflow for use with poultry, which begins with placing a pre-specified weight of raw poultry into a Pathotrak bag equipped with a capture membrane. Then, 300 mL of a carrier fluid is introduced to the bag to facilitate release of the bacteria from the sample and the transfer of those bacteria to the capture membrane. The bag is subjected to isostatic pressure, which forces the sample through the capture membrane and separates the pathogens from the food matrix. The captured pathogens in the NGE concentrator are then eluted with 1 mL PBS, (Sigma-Aldrich, St. Louis, MO, USA), concentrated via centrifugation to a target volume of 20 µL, and prepared for molecular detection using the Neogen Molecular Detection System (MDS), (Neogen Corp, Lansing, MI, USA).

### 2.1. Preparation of Chicken Samples and Pathogen Cultures

#### 2.1.1. Chicken Sample Preparation

Boneless, skinless chicken breasts were obtained from a local retailer and cut into roughly equal-sized fragments. A total of 325 g of chicken was transferred into 500 µm stomacher filter bags nested within Pathotrak Next Generation Enrichment (NGE) bags, (Pathotrak Inc., Laurel, MD, USA). These bags were securely sealed using a heat sealer to prevent leakage during subsequent handling.

#### 2.1.2. Pathogen Cultures

Pathogen cultures were prepared using single colonies of each strain inoculated into appropriate media. *Escherichia coli* O157:H7 (strain 20R2R) and *Salmonella enterica* subsp. *enterica* serovar Newport (strain SN11/00) were grown in Luria–Bertani (LB) media, (Becton Dickinson, and Co., Sparks, MD, USA). *Listeria monocytogenes* (strain ATCC 19111) was cultured in Brain Heart Infusion (BHI) broth, (Becton Dickinson, and Co., Sparks, MD, USA), and *Campylobacter jejuni* (strain S27) was grown in Brucella broth, (Becton Dickinson, and Co., Sparks, MD, USA). All cultures were incubated overnight at 37 °C with shaking at 170 RPM, except for *Campylobacter*, which was incubated statically at 42 °C in an airtight biotransport box with a BD Campy GasPak, (Becton Dickinson, and Co., Sparks, MD, USA) under microaerobic conditions.

#### 2.1.3. Preparation of Chicken Rinse for Standard Curve Experiments

A total of 325 g of chicken and 300 mL of BPW, (Bio-Rad, Hercules, CA, USA) were transferred into stomacher bags containing 500 µm filter bags. Samples were homogenized in the Stomacher 3500 (Seward Laboratory Systems Inc. West Sussex, UK) for 30 s at the “normal” setting. The liquid was removed from the filtered section of the stomacher bag to exclude particles greater than 500 µm.

#### 2.1.4. Preparation of Serial Dilutions of Bacterial Cultures

Serial dilutions were prepared to establish standard curves for the MDA assay. Seven 10-fold serial dilutions ranging from 10^8^ to 10^2^ CFU/mL were prepared for each pathogen in chicken meat rinse. For inoculation of meat samples, the serial dilution was performed in meat rinse. Specifically, 0.5 mL of overnight culture was combined with 4.5 mL of meat rinse, vortexed, and serially diluted through six additional steps. Note, the samples used to generate the standard curve were generated by spiking known quantities (determined by 6 × 6 plating) of pathogens into the meat rinse to provide an estimate of the response for the specific concentration of pathogen in a manner that accounted for matrix inhibition.

### 2.2. Preparation for Pathotrak–MDS Detection Experiment

To simulate contamination, chicken samples were inoculated with pathogen cocktails at concentrations of 5 CFU/g or 25 CFU/g. For the 5 CFU/g concentration, 162.5 µL of pathogen cocktail containing a target concentration of 10^4^ CFU/mL each of *Salmonella*, *Listeria*, *Campylobacter*, and *E. coli* O157 strains was added to 325 g of chicken. For the 25 CFU/g concentration, 81.25 µL of a 10^5^ CFU/mL cocktail was used. Uninoculated samples served as negative controls.

### 2.3. Pathotrak Process

The Pathotrak Next Generation Enrichment (NGE) process, as illustrated in Figure 1, involves four key stages: pressure cycle, elution cycle, centrifugation cycle, and automated pipettor stage. To extract and concentrate pathogens, 300 mL of Pathotrak buffer, (Pathotrak Inc., Laurel, MD, USA) was added to the Pathotrak NGE bags containing chicken samples. The bags were heat sealed and then gently mixed for five minutes to wet the surface of the chicken before undergoing a 10-min pressure cycle in the Pathotrak chamber (Pathotrak Inc., Laurel, MD, USA).

After the pressure cycle, the NGE-EXTR-3 filters (Pathotrak Inc., Laurel MD lot PEC322081400) were eluted using a 3 mL syringe containing 1 mL of PBS buffer. Eluted samples were centrifuged in Eppendorf 5424, (Eppendorf, Hamburg, Germany) at 12,000× *g* for two minutes. The supernatant volume was reduced to approximately 20 µL using the automated Pathotrak pipettor (Pathotrak Inc., Laurel, MD, USA), with each cycle lasting 3 min.

#### Elution Volume Measurement

The final elution volume was estimated using a micropipette. The volume of the pipette was set to 12 µL, ~8 µL less than the projected final volume. Following a 5 s quick spin in a mini centrifuge to collect all the liquid in the bottom of the tube, the liquid was aspirated with a pipette. With the pipet tip submerged, the dial on the pipet was turned until the entire volume was removed from the tube. The pipet dial reading was recorded as the final volume.

### 2.4. Molecular Detection System (MDS) Assays

A multiplex format depicted in Figure 1 was used to detect *Salmonella*, *Listeria*, *Listeria monocytogenes*, *Campylobacter*, and *E. coli* O157. For both standard curve and detection samples, the entire 20 µL resuspended, concentrated, eluate from each sample was mixed with lysis reagent. Note, while the lysis reagents are interchangeable between kits, the lysis reagent from the Molecular Detection Assay 2 Listeria monocytogenes kit, (Neogen Corp, Lansing, MI, USA) was used for these experiments. Samples were heated to 100 °C for 15 min, cooled to room temperature, and 20 µL of lysate was transferred to Molecular Detection System Assay 2 reagent tubes for *Campylobacter* (CAM), *Salmonella* (SAL), *Listeria monocytogenes* (LMO), *Listeria* (LIS), and *E. coli* O157 (including H7) (ECO) (Neogen Corp, Lansing, MI, USA) and processed as per the manufacturer’s instructions for the detection of the specified bacteria.

### 2.5. Plating to Determine Standard Curve Concentration

The 6 × 6 drop plate assay was used to determine the actual concentration (CFU/mL) of the starting inoculum for each pathogen in the standard curve. LB agar was used for plating *Salmonella* and *E. coli*, BHI agar for *Listeria*, and Brucella agar for *Campylobacter*. Plates were incubated using an Innova 4200 incubator, (New Brunswick Scientific, Edison, NJ, USA) under conditions appropriate for each pathogen. The measured concentration of the respective inoculum was used to calculate the theoretical values with which the samples were contaminated.

### 2.6. Data Collection and Analysis

Data from LAMP assays were exported from Molecular Detection Software version 2.3.0.1, (Neogen Corp, Lansing, MI, USA). The exported run report provides the prevalence response (positive/negative) and measured response (RLUs) vs. time curve for each well. These results were used to construct standard curves and determine detection limits for the 5 and 25 CFU/g concentrations. Colony counts from plating were analyzed to confirm pathogen presence and viability.

### 2.7. Analysis of MDS Results

**Figure A1:**
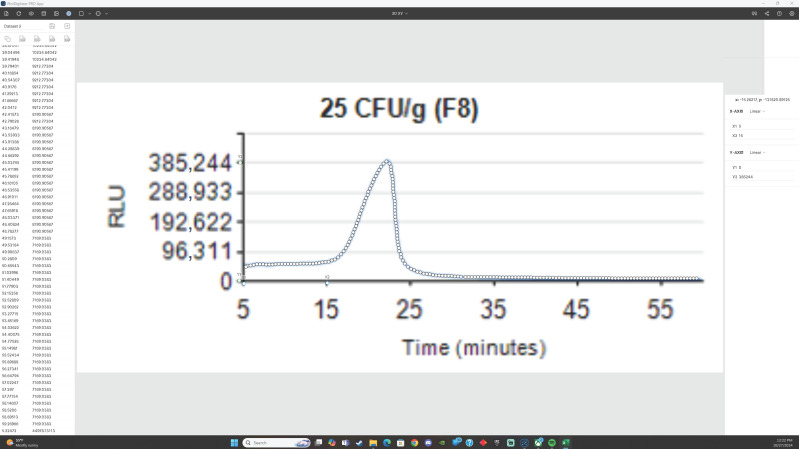
Screen capture from PlotDigitizer Pro. The screen capture showcases the raw MDS graph overlaid with discrete data points represented as blue dots. The corresponding x and y values for each data point are displayed in the table on the left, formatted for easy export and further mathematical analysis.

The MDS software produced visual output in the form of graphs showing relative light units (RLUs) versus time (minutes) for each pathogen at varying concentrations. The graphs exported by MDS software version 2.3.0.1 (Neogen Corp, Lansing, MI, USA) were image-based, necessitating digitization to quantitatively determine the time to detection (TTD). Using PlotDigitizer Pro version 2.6.9, the RLUs versus time curves were digitized to extract precise data points for further analysis. The digitized data enabled the conversion of visual trends into quantitative metrics for TTD. The appendix includes Figure A1, which showcases the overlay curves from the MDS, illustrating how the raw image data were digitized to produce the quantitative results that informed the standard curve analysis.

To analyze the digitized data, a four-parameter logistic (4PL) regression model was applied. This model, particularly suited for analysis of sigmoidal response curves, was defined by four key parameters: the minimum response (baseline), the maximum response (plateau), the inflection point (time at which the signal reaches half of its maximum and serves as the critical TTD metric), and the slope (indicating the steepness of the response). All the quantitative data was analyzed using JMP software version 16.2.0.

## 3. Results

### 3.1. Efficiency of Pressure Cycle

The efficiency of the pressure cycle within the Pathotrak process was evaluated by measuring the percentage of retained weight in the bags after the cycle. This calculation was performed using the formula:

(1) $$\%\;retained\;weight=\frac{final-initial}{initial}$$

Chicken samples combined with Pathotrak buffer in NGE bags were pressurized in the Pathotrak pod. The objective was to pass all fluid completely through the NGE filters, leaving no net residual fluid. Observations confirmed that no clogging occurred during the cycle, the bags appeared visibly dry, and the bag that was compressed appears similar to when chicken breasts are vacuum-sealed. However, a small amount of retained weight was recorded, indicative of residual material that remained within the bags. These results indicate that while the pressure cycle effectively displaces most of the buffer from the bags, a small percentage of weight is retained. This retention may be due to minor residual material clinging to the bag’s interior or onto the surface of the chicken.

**Figure 2:**
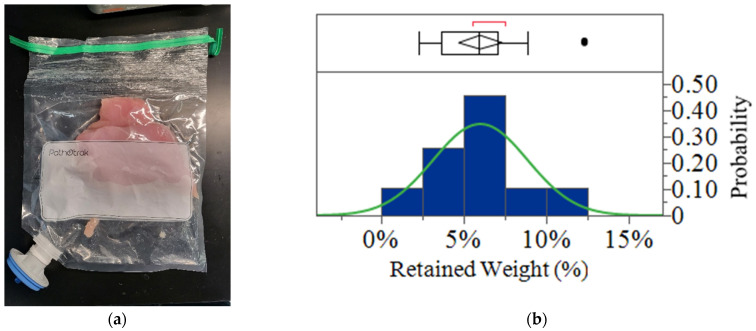
Post-pressure cycle analysis. (**a**) A photograph of the chicken sample within the Pathotrak bag after the pressure cycle, illustrating the vacuum-sealed appearance of the bag. (**b**) Histogram (blue) and fitted normal distribution curve (green) of the percentage of retained material in the bag post-pressure cycle. The *y* axis represents the probability density of retention percentages. The overlaid box plot summarizes key distribution statistics, including the median, interquartile range (IQR), whiskers, potential outliers (black circles), and the 95% confidence interval for the mean (diamond). The red bracket highlights the densest 50% of observations.

Figure 2a shows a sample of the chicken within a Pathotrak bag post-cycle. The distribution of the retained weight percentages across all samples is depicted in Figure 2b. The median percentage of retained weight was found to be 6%, with an interquartile range (IQR) between 4% and 7%. The minimum observed retention was 2%, while the maximum reached 12%, suggesting a consistent but slight residual presence across samples. The mean retained weight was calculated as 6% (Std Dev = 3%). The distribution fitted a normal curve, supported by a Shapiro–Wilk test (W = 0.907 and *p* = 0.0553) and an Anderson–Darling test (A2 = 0.579 and simulated *p* value = 0.1168).

### 3.2. Evaluation of Automated Pipettor Consistency Versus Manual Elution

**Figure 3:**
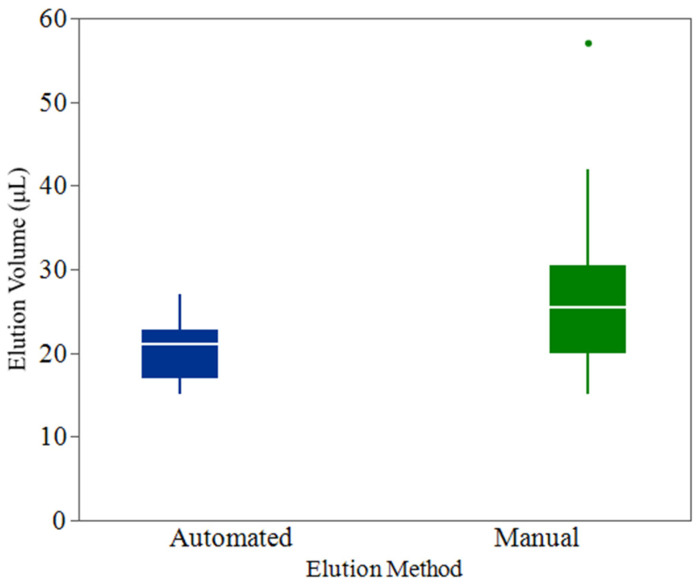
Box plot comparison of elution volumes between automated and manual methods. The boxes represent the interquartile range (IQR), with the horizontal line indicating the median elution volume. Whiskers extend to the minimum and maximum observed values within 1.5 times the IQR, with any data points beyond marked as outliers. The automated method demonstrated significantly lower and more consistent elution volumes compared to the manual method (*p* < 0.0001), with tighter 95% confidence intervals around the mean. Points beyond the whiskers of the box plots represent outliers.

The automated pipettor’s efficiency in reducing elution volumes to approximately 20 µL was compared to the manual method. The data, presented in Figure 3, highlight the volume distribution differences between these two approaches.

The automated method achieved a mean elution volume of 20.4 µL (Std Dev = 3.6 µL) across 48 replicates. The distribution showed a narrow interquartile range (IQR) from 17 µL to 22.8 µL, with a median of 21 µL. The minimum observed volume was 15 µL, and the maximum was 27 µL. The 95% confidence interval for the mean volume was between 19.4 µL and 21.5 µL, indicating the automated pipettor’s consistent performance around the target volume. A one-sample t-test was performed to compare the mean elution volume against the hypothesized target of 20 µL, yielding a test statistic of 0.85 and a *p* value of 0.398. This result suggests no significant deviation of the automated method’s mean from the target, underscoring its reliability.

In contrast, the manual method displayed a higher mean elution volume of 27.8 µL (Std Dev = 10.6 µL), with a broader IQR ranging from 20 µL to 34 µL and a median of 25.5 µL. The elution volumes ranged from 15 µL to 57 µL, demonstrating a greater spread and higher variability. The 95% confidence interval for the mean elution volume was 24.8 µL to 30.9 µL, highlighting the inconsistent nature of manual pipetting. The one-sample t-test comparing the manual method’s mean to the 20 µL target produced a test statistic of 5.1 and a *p* value of <0.0001, indicating a statistically significant difference. This result confirms that the manual method’s mean elution volume was substantially higher than the desired target, which could impact the uniformity and precision required for downstream processes.

### 3.3. Development of a Standard Curve for Time to Detection (TTD) Using MDS Output

To construct a standard curve for pathogen detection, data were collected using the Neogen Molecular Detection System (MDS), a LAMP-based assay platform that continuously monitors relative luminescence units (RLUs) over time. Similar to cycle threshold (Ct) values in qPCR, time to detection (TTD) reflects the time it takes for the detection signal to reach a meaningful threshold, correlating with the concentration of the target pathogen in the sample.

**Figure 4:**
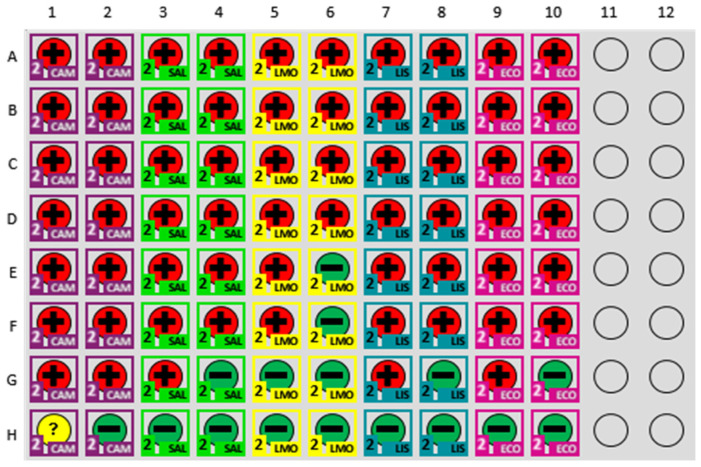
MDS Plate Layout for Standard Curve Development. MDS plate layout and prevalence output used for generating the standard curve for time to detection. Each row represents a specific dilution level of a pathogen, with rows A–G depicting 10^8^ to 10^2^ CFU/mL and row H serving as negative controls. The plate includes duplicates for each dilution to ensure accuracy and reliability of the detection results. The color-coded representation indicates the type of pathogen tested (CAM = *Campylobacter* spp., SAL = *Salmonella*, LMO = *Listeria monocytogenes*, LIS = *Listeria* spp., and ECO = *Escherichia coli* O157). Positive results are reported in red (+), and negative in green (–). Indeterminate results are reported in yellow (?).

The experimental plate layout is depicted in Figure 4 and details the arrangement used for the assays. Each row (A–G) represented a 10-fold dilution series, ranging from 10^8^ to 10^2^ CFU/mL, with duplicate wells per concentration to enhance result reliability. Row H contained negative control wells to confirm assay specificity. Pathogens tested included *Campylobacter* spp., *Salmonella*, *L. monocytogenes*, *Listeria* spp., and *E. coli* O157. The MDS output confirmed consistent detection at higher concentrations, while at lower concentrations some wells showed false negatives, highlighting assay limitations in sensitivity at the lower limit of detection.

**Figure 5:**
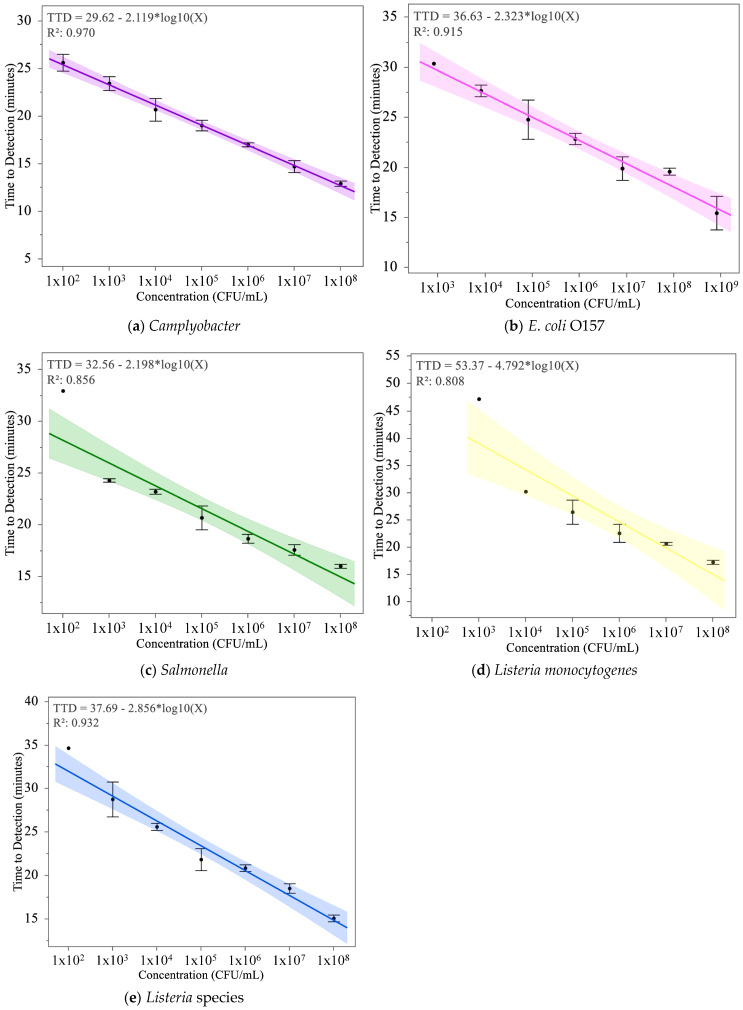
Standard Curves for Pathogen Detection Assays. The standard curves for five pathogen detection assays, plotting time-to-detection (TTD) in minutes against the concentration of bacteria (CFU/mL) on a log scale. The regression equation and R-squared values are included for each assay, indicating the relationship between bacterial concentration and TTD. The shaded regions represent the 95% confidence interval for the mean, highlighting the precision of the regression model across tested concentrations. Error bars denote the standard deviation of the mean for each data point. The lines are color-coded to correspond with the colors used by Neogen to identify each pathogen kit: *Campylobacter* ((**a**), purple), *E. coli* ((**b**), pink), *Salmonella* ((**c**), green), *Listeria monocytogenes* ((**d**), yellow), and *Listeria* species ((**e**), blue). These curves provide a comparative visualization of the performance and variability of the assays under the tested conditions.

The analysis of standard curves for each tested pathogen revealed distinct characteristics and variabilities in TTD performance across different concentrations, as presented in Figure 5. The results of the standard curves for the five pathogen detection kits, summarized in Table 1, highlight key parameters, including R-squared values, slopes, intercepts, root mean square error (RMSE), and the highest concentrations at which false negatives were observed. These parameters provide critical insights into the linearity, sensitivity, reliability, and overall performance of the assays.

The R-squared values, which represent the proportion of variance in TTD explained by the log-transformed concentration, ranged from 0.808 for *L. monocytogenes* to 0.970 for *Campylobacter*. The *Campylobacter* assay exhibited the strongest linearity, emphasizing its consistent performance across the tested concentration range. In contrast, the *L. monocytogenes* assay showed the lowest R-squared value, reflecting increased variability in detection times, particularly at lower concentrations. The RMSE values further quantify variability, with *Campylobacter* having the lowest RMSE (0.808), confirming its minimal deviations from the regression model, while *L. monocytogenes* exhibited the highest RMSE (4.118), indicating significant variability.

Slopes and intercepts, derived from the TTD equations in the graphs, provide additional insights into the assays’ performance. The slope, representing the rate of change in TTD with respect to log_10_ concentration, was steepest for *L. monocytogenes* (−4.792), indicating a rapid decrease in TTD as concentration increased. This sensitivity at higher concentrations, however, corresponded to greater variability at the lower detection limit, as indicated by false negatives observed at10^4^, 10^3^, and 10^2^ CFU/mL. Conversely, *Campylobacter* had the most moderate slope (−2.119), which resulted in reliable performance across the tested range without any false negatives.

Intercept values, which represent the theoretical TTD at a log_10_ concentration of 0, ranged from 29.62 for *Campylobacter* to 53.37 for *L. monocytogenes*. Higher intercepts, such as those for *L. monocytogenes* (53.37) and *Listeria* spp. (37.69), reflect longer TTDs overall, particularly at lower concentrations, where reduced sensitivity was observed. Lower intercepts, such as those for *Campylobacter* (29.62) and *Salmonella* (32.56), correspond to shorter TTDs, supporting their applicability for detecting pathogens at lower bacterial loads. These trends were statistically significant for all pathogens, as indicated by *p* values for the regression models (*p* < 0.0001 or *p* = 0.0004).

False negative rates provided additional insight into assay performance at the lower detection range. The *Campylobacter* assay was the only kit with no false negatives across the tested concentrations, emphasizing its robustness. In contrast, false negatives were observed at 10^2^ CFU/mL for *Salmonella*, *Listeria* spp., and *E. coli*, and at multiple concentrations (10^4^, 10^3^, and 10^2^ CFU/mL) for *L. monocytogenes*, indicating the assays’ diminished reliability at low bacterial loads. These findings suggest that while the *L. monocytogenes* assay is highly responsive to higher concentrations, its sensitivity at the lower end may require further optimization.

### 3.4. Semiquantitative Assessment of MDS Output from Artificially Inoculated Chicken Samples

The Pathotrak protocol was employed to assess the capability of detecting multiple bacteria, *Campylobacter*, *Salmonella*, *E. coli*, *L. monocytogenes*, and general *Listeria* spp., in chicken breast samples inoculated at 5 and 25 CFU/g. This study was conducted across three trials, with each trial involving three chicken samples inoculated at 5 CFU/g (rows A, B, and C), three samples at 25 CFU/g (rows D, E, and F), positive control samples containing a mixture of all pathogens at 10^6^ CFU/mL (row G), and negative control samples comprising uninoculated chicken (row H).

**Figure 6:**
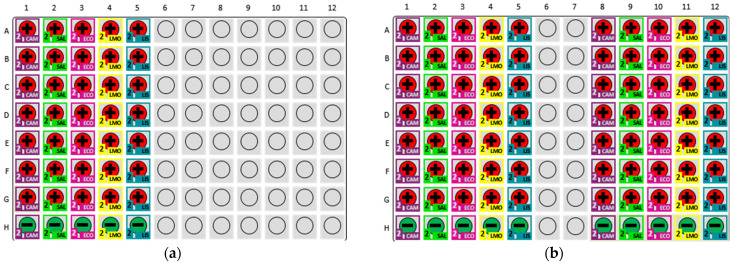
MDS output showcasing the results of pathogen detection in chicken samples inoculated at 5 and 25 CFU/g across three trials. Positive results are reported in red (+), and negative in green (–). (**a**) Trial 1 output shows positive detection for all tested pathogens in inoculated samples, while negative controls remain pathogen-free. (**b**) Combined output for Trials 2 and 3 confirms consistent detection across all inoculated samples, with negative controls maintaining no detection. These results highlight the Pathotrak protocol’s compatibility with multiplex detection, enabling the concurrent identification of multiple pathogens.

Figure 6 presents the MDS output for these trials. In all three trials, the MDS system successfully detected all five bacteria in each of the 5 and 25 CFU/g inoculated chicken samples. This consistent detection underscores the ability of samples prepared with the Pathotrak to be effectively processed with multiplex detection methods, enabling simultaneous identification of multiple pathogens in complex matrices such as chicken meat. The negative control samples consistently showed no detection across all trials, confirming the specificity of the assay and affirming that the chicken used was pathogen-free or contained pathogen levels below the assay’s detection threshold.

The detection data from chicken samples inoculated with 5 or 25 CFU/g of *Campylobacter*, *Salmonella*, *E. coli*, and *L. monocytogenes* were analyzed to evaluate the performance of multiplexed detection using MDS kits. These kits targeted *Campylobacter*, *Salmonella*, *E. coli* O157 (including H7), *Listeria* spp., and *L. monocytogenes*. TTD data were derived by digitizing the MDS output graphs. A 4PL regression model was applied to the TTD data, enabling the calculation of MDS-measured concentrations using the standard curves developed for each pathogen kit. These digitized detection curves can be found in Appendix B.

**Figure 7:**
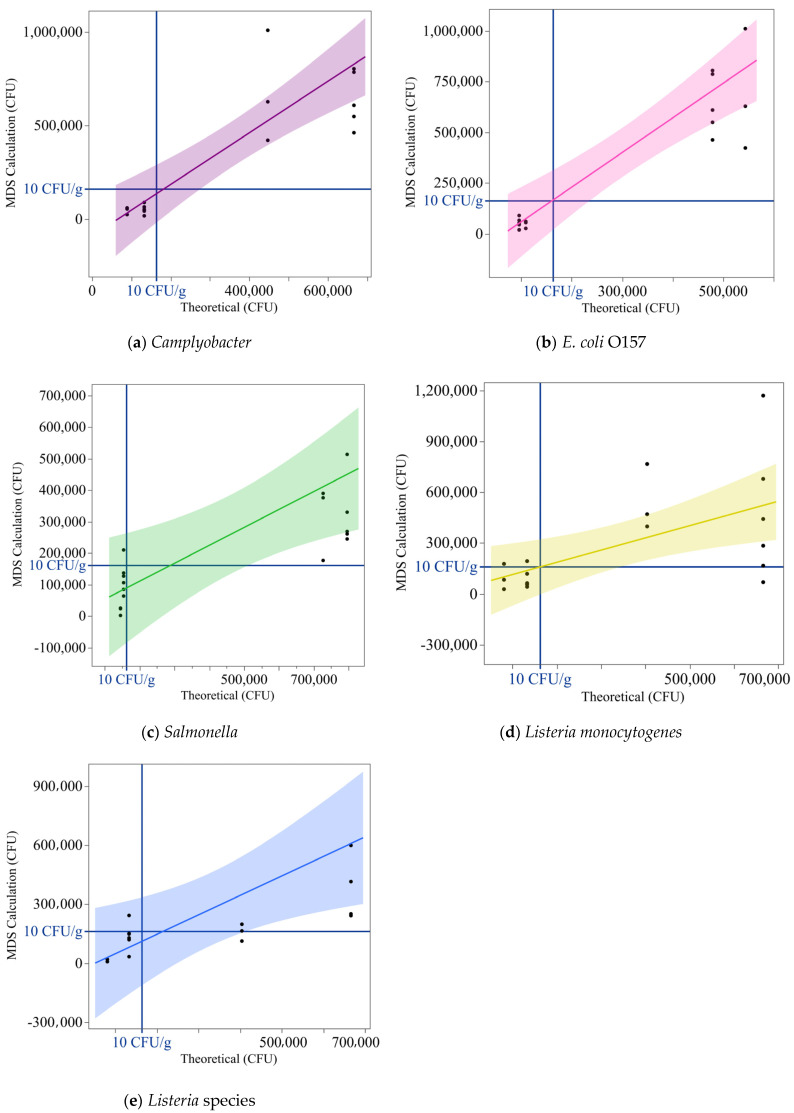
Theoretical vs. Measured Pathogen Concentrations Using MDS Kits post processing with the Pathotrak NGE. Theoretical concentrations (CFU/g) are plotted on the *x* axis against MDS-measured concentrations (CFU/g) on the *y* axis for bacterial pathogen assays: *Campylobacter*, *E. coli* O157, *Salmonella*, *Listeria monocytogenes*, and *Listeria* species. The dark blue horizontal and vertical lines indicate the critical detection threshold of 10 CFU/g. Each data point represents the relationship between the theoretical and measured concentrations for an individual sample. The colored regression lines, matching the colors used in the MDS kits for each pathogen, illustrate the fit through the data points. Shaded regions around the regression lines represent the 95% confidence intervals, highlighting variability in the detection measurements. Panels are color-coded as follows: purple for *Campylobacter* (**a**), pink for *E. coli* O157 (**b**), green for *Salmonella* (**c**), yellow for *Listeria monocytogenes* (**d**), and blue for *Listeria* species (**e**).

To ensure accuracy, the theoretical concentration for each sample was determined by 6 × 6 plating the cultures used to inoculate the samples. The MDS-measured concentrations were then plotted against these theoretical values, with a critical threshold of 10 CFU/g, highlighted by blue reference lines (Figure 7). Each point represents the number of cells inoculated on the meat, estimated from the 6 × 6 drop plate assay against the number of cells predicted by the MDS. This threshold served as a key benchmark: samples inoculated with 5 CFU/g were expected to yield measured concentrations below 10 CFU/g, while those inoculated with 25 CFU/g were expected to exceed this threshold. The plot was divided into four quadrants to interpret the detection outcomes relative to the critical threshold.

Data points in the bottom left quadrant represented true negatives, where measured concentrations correctly fell below 10 CFU/g. Points in the top left quadrant indicated false positives, where the system incorrectly measured concentrations above the threshold. Data points in the top right quadrant corresponded to true positives, with concentrations accurately identified as above 10 CFU/g. Finally, points in the bottom right quadrant represented false negatives, where the system failed to detect concentrations exceeding the critical threshold. The regression line, derived from the relationship between measured and theoretical concentrations, was overlaid on the plot. The shaded region around the regression line represented the 95% confidence interval, providing a visual depiction of the data’s variability and the system’s precision.

The detection capabilities of the MDS system in combination with the Pathotrak NGE for *Campylobacter*, *Salmonella*, *L. monocytogenes*, *Listeria* spp., and *E. coli* O157 were evaluated through regression and contingency analyses. Figure 7a–e provide detailed visualizations of the relationship between theoretical inoculation levels and MDS-calculated concentrations, as well as the agreement between predicted and actual results. Overall, the MDS system exhibited strong detection capabilities across all pathogens, with variability in sensitivity and precision influenced by pathogen type and concentration. Statistical tests, including chi-square, Fisher’s exact test, and Bowker’s test, were used to quantify the reliability of the measurements.

The analysis for *Campylobacter* (Figure 7a) and *E. coli* (Figure 7b) revealed high accuracy and strong predictive performance. Both pathogens exhibited minimal variability and tight alignment of data points along the regression lines. For *Campylobacter*, no false positives or false negatives were observed, and the system achieved a Kappa coefficient of 1. Statistical tests, including the likelihood ratio and Pearson chi-square, were highly significant (*p* < 0.0001). Similarly, *E. coli* detection showed agreement, with no false classifications and a Kappa coefficient of 1. Fisher’s exact test and Bowker’s test further corroborated the absence of variability or misclassification for both pathogens. These results confirm the robustness and reliability of the detection system for these pathogens.

The analyses for *Salmonella* revealed intermediate detection performance. For *Salmonella*, variability was observed near the detection threshold, with one false positive (measured to be >10 CFU/g) identified for a sample inoculated at 5 CFU/g (Figure 7c). The Kappa coefficient of 0.889 reflected agreement overall, although this misclassification reduced the overall reliability. Statistical tests, including likelihood ratio chi-square and Pearson chi-square (*p* < 0.0001), confirmed the significance of these results. Fisher’s exact test and the Cochran–Armitage trend test highlighted the system’s overall capability while emphasizing reduced sensitivity near the critical threshold.

Similarly, the detection of *Listeria* spp. showed intermediate performance, with one false positive and one false negative observed (Figure 7e). The regression analysis revealed variability at higher concentrations, with a Kappa coefficient of 0.778 indicating agreement. Statistical tests, including likelihood ratio chi-square (*p* = 0.0004) and Pearson chi-square (*p* = 0.0010), confirmed the significance of these findings. Fisher’s exact test and Bowker’s test further emphasized the detection system’s reliability while highlighting the need for sensitivity improvements near the detection threshold.

For *L. monocytogenes*, the detection system exhibited the highest variability, with two false positives and two false negatives observed (Figure 7d). The regression analysis showed a moderate correlation between theoretical and MDS-calculated concentrations, with a Kappa coefficient of 0.5556 indicating the least agreement among the pathogens tested. However, the statistical tests, including likelihood ratio chi-square (*p* = 0.0153) and Pearson chi-square (*p* = 0.0184), confirmed that the agreement was non-random. Fisher’s exact test corroborated these findings, though Bowker’s test and the observed misclassifications underscored the challenges of detecting *L. monocytogenes* at concentrations near the critical threshold. These results emphasize the need for assay optimization to enhance sensitivity and reduce variability in detection performance.

## 4. Discussion

This study highlights the potential of the Pathotrak process, in combination with the Neogen Molecular Detection System (MDS), to streamline multiplex pathogen detection in food samples. By coupling efficient sample preparation with robust detection methodologies, the system provides a promising approach for both qualitative and semiquantitative analysis of key foodborne pathogens, including *Campylobacter*, *E. coli* O157, *Salmonella*, *Listeria* spp., and *L. monocytogenes*.

### 4.1. Standard Curve

When comparing the standard curves across the different pathogens, several key trends emerged. All assays showed a negative correlation between pathogen concentration and TTD, confirming that higher concentrations resulted in faster detection. However, the slope and intercept values varied, indicating differences in assay sensitivity and consistency.

While the MDS system primarily provides qualitative positive/negative responses, its time-to-detection (TTD) metric offers additional insights into pathogen concentration. The software will report positive wells in real time as the LAMP assay reaches the detection threshold, while negative samples are not reported until the assay completes its full duration. This distinction in reporting emphasizes the binary nature of MDS outputs but also provided the insight to apply TTD for semiquantitative analysis.

The RLUs vs. time were digitized, and the TTD was extracted using a 4PL regression model. This method provided the framework for analyzing detection trends across pathogens. The TTD metric appears to be a reliable indicator of pathogen concentration, with regression models yielding strong linear relationships for most pathogens. Comparative analysis of standard curves revealed distinct detection dynamics across pathogens.

*Campylobacter* demonstrated the highest consistency, with an R-squared value of 0.970, reflecting reliable detection across the entire concentration range with minimal variability. No false positives were observed, although Figure 4 presents one flagged retest for the negative control. Upon retesting, the sample was correctly classified as negative, indicating the assay’s accuracy in resolving potential ambiguities. These findings highlight the robustness of the *Campylobacter* assay and the MDS system’s ability to handle low pathogen loads.

In contrast, the *L. monocytogenes* kit exhibited the greatest variability, with an R-squared value of 0.808 and the steepest slope among tested pathogens, indicative of high sensitivity at higher concentrations but substantial inconsistencies at lower levels. Notably, Figure 4 indicates this assay exhibited false negatives at concentrations as high as 10^4^ CFU/mL, underscoring the assay’s sensitivity challenges near the detection threshold. Similarly, assays for *Salmonella* and *E. coli* displayed reduced reliability at low concentrations, with both assays exhibiting false negatives at 100 CFU/mL. For the *Listeria* kit the assay demonstrated better performance than *L. monocytogenes* (R-squared = 0.932), though its steeper slope suggested potential over-sensitivity to concentration changes, potentially limiting its consistency near the detection limit.

These findings emphasize the variability in pathogen-specific detection reliability and the need to address the influence of the MDS system’s limit of detection on assay performance. While the standard curve analysis validated the system’s robustness at higher concentrations, broader confidence intervals and false negatives at lower levels highlight the importance of optimizing detection capabilities near the detection threshold.

Given these uncertainties, future work is needed to systematically evaluate the effects of lysis efficiency, target abundance, inhibition, and background microbial interactions on detection sensitivity [18,19,20,21,22]. Without further empirical investigation, any conclusions regarding these factors remain speculative. Note, the absence of reported internal amplification controls (IAC) within the MDS is a limitation, however, the assays have undergone validation and a method certified according to ISO 16140-2 standards. Additional studies could focus on directly quantifying lysis efficiency across different bacterial species, assessing the results relative to the precise genomic targets used in the MDS assays, and identifying if any specific inhibitors impact the detection differences. Such investigations would provide a clearer understanding of the factors influencing assay performance and help refine molecular detection strategies for foodborne pathogens.

### 4.2. Semiquantitative Detection of Pathogens in Inoculated Chicken Samples

The application of the Pathotrak process to poultry meat samples inoculated at 5 and 25 CFU/g demonstrated the system’s practical utility to provide semiquantitative results without enrichment. The Pathotrak pressure cycle was a critical component of the sample preparation process, efficiently removing buffer fluid from samples and leaving only minimal residual material. The observed median retained weight of 6% (IQR: 4–7%) suggests a high level of consistency in the process, with retained material likely attributing to minor residuals clinging to the interior of the bag or chicken surface. This reliable performance minimizes variability during sample preparation, ensuring that downstream detection is not compromised. Similarly, the automated pipettor provided a clear advantage over manual methods by achieving precise and consistent elution volumes. The narrow interquartile range (IQR: 17–22.8 µL) and lack of significant deviation from the target volume of 20 µL (*p* = 0.398) emphasize the importance of automation in reducing operator variability and improving reproducibility.

The Pathotrak protocol effectively concentrated pathogens in complex matrices, enabling their detection by the MDS system. Figure 6 shows that all pathogens were consistently detected at both concentrations tested across multiple trials, reflecting the reliability of the combined system. When comparing the MDS-calculated concentrations with theoretical inoculation levels, the data provided further insights into the system’s strengths and limitations.

The assay was able to consistently distinguish between higher than and lower than 10 CFU/g levels of contamination from *Campylobacter* and *E. coli* when inoculated at 5 and 25 CFU/g. As shown in Figure 7a,b, no false positives or false negatives were observed, reflecting the assay’s robustness in detecting these pathogens even at low levels. *Salmonella* detection exhibited more variability, particularly at 5 CFU/g. Figure 7c indicates consistent detection was achieved at 25 CFU/g, however, the occurrence of a false positive at 5 CFU/g indicates limitations at lower inoculation levels.

The results for *Listeria* spp. (Figure 7e) exhibited more variability as there was a false positive response at 5 CFU/g and a false negative at 25CFU/g. *Listeria monocytogenes* presented the greatest challenges in detection reliability. Figure 7d indicates that detection was sporadic, reflecting substantial variability in assay sensitivity at 25 CFU/g and 5 CFU/g. It is unclear why the *Listeria* (LIS) and *Listeria monocytogenes* (LMO) kits showed different assay sensitivity, but this is likely due to different primers used in the LIS vs. LMO assays.

The observed differences in detection efficiency between *Campylobacter*, *E. coli*, and *Listeria* could be influenced by pathogen-specific factors such as bacterial adhesion properties, and interactions with the food matrix. Studies have shown that bacterial attachment mechanisms vary significantly depending on both the food matrix and microbial species [23,24,25]. These differences may explain why detection efficiency varied among the pathogens tested, particularly for *Listeria monocytogenes*, which exhibited the greatest inconsistency in assay performance.

It is also important to clarify that the combined Pathotrak–MDS workflow represents a fundamentally different detection approach compared to traditional culture-based methods. Standard enrichment techniques rely on overnight incubation to increase bacterial populations before detection, while the Pathotrak system eliminates the need for this step, reducing the total time to results to under three hours for 96 samples. This workflow offers several advantages, including the elimination of pathogen-specific enrichment media, a reduction in the time required for incubation, and a minimizing of the hands-on sample processing time. By streamlining the pathogen detection workflow, the system enables faster decision-making for food safety applications. Additionally, because it does not rely on bacterial growth, this method has the potential to detect viable-but-non-culturable (VBNC) organisms, which would otherwise be undetectable using traditional plating methods. This may include VBNC organisms as well as organisms that may no longer be culturable but remain intact and, thus, are retained by the Pathotrak capture membrane.

Despite these advantages, this approach should not be viewed as a direct substitute for culture-based methods, but rather as an alternative detection strategy. While molecular methods allow for rapid pathogen screening, they do not recover viable isolates, which may be necessary for further characterization in regulatory or epidemiological investigations.

## 5. Conclusions

The combined Pathotrak–MDS workflow demonstrates several advantages when considered holistically. Its enrichment-free approach significantly reduces detection times while maintaining accuracy, making it well-suited for applications requiring rapid decision-making. By eliminating pathogen-specific enrichment steps, the protocol streamlines workflows, minimizes resource use, and reduces operator variability, while also allowing simultaneous detection of multiple pathogens. This ability to multiplex enhances its utility in both regulatory and industrial settings, where efficiency and throughput are critical. The system’s consistent detection of all pathogens at 5 and 25 CFU/g reinforces its robustness for moderate contamination levels, highlighting its potential for broader adoption. However, variability in the MDS results observed at lower concentrations underscores the need for further refinement to improve sensitivity near detection limits, particularly for pathogens subject to zero-tolerance policies, such as *L. monocytogenes* and Shiga toxin-producing *E. coli*.

The ability to correlate TTD values with pathogen concentrations offers an opportunity for semiquantitative applications. By leveraging the relationship between TTD and pathogen levels, as established through standard curves, the system could be extended beyond binary prevalence outcomes to provide semiquantitative estimates of contamination. This capability is particularly valuable for pathogens like *Campylobacter* and *Salmonella*, where regulatory thresholds rather than zero tolerance apply. The Pathotrak–MDS workflow presents an opportunity to unify poultry testing methods for different pathogens, which are currently conducted separately using MLG 4.15 and MLG 41.09, respectively.

Nonetheless, despite its potential, the system faces challenges for zero-tolerance pathogens, where regulatory compliance demands no false negatives. During this study, false negatives were observed in the development of standard curves for the MDS assays, suggesting that further methodological refinement is needed for such applications. Refining the cell lysis and dilution steps prior to MDS testing could reduce subsampling variability and improve the limit of detection. Potential improvements include adjusting the dilution factor during lysis or increasing the volume transferred to reagent wells to enhance sensitivity. Furthermore, increasing the final Pathotrak elution volume from 20 µL to 50 µL would provide two key advantages: allocating a portion for molecular screening while reserving the remainder for culture-based assays, enabling isolate recovery for regulatory compliance and further characterization.

These findings highlight the potential of the Pathotrak–MDS workflow to transform multiplex pathogen detection in poultry samples. Addressing current limitations and refining assay sensitivity will be essential to further enhance the system’s reliability. While this study demonstrates the feasibility of using the MDS response as a semiquantitative assay to determine whether contamination exceeds a threshold, additional research is needed to validate the assay’s repeatability and robustness. Integrating TTD-based insights with efficient sample preparation protocols could improve risk assessments and regulatory decision-making. These advancements could represent a significant step toward faster, more accurate pathogen detection, ensuring safer food supply chains while meeting the stringent demands of modern food safety standards.

## Figures and Tables

**Table 1:** Key Parameters of Standard Curves for Pathogen Detection Assays. This table summarizes the regression parameters (R-squared value, root mean square error, slope, and intercept) for standard curves developed for five pathogen detection assays: *Campylobacter*, *Salmonella*, *E. coli*, *Listeria* species, and *Listeria* monocytogenes. It also highlights the highest concentrations at which false negatives were observed, providing an overview of each assay’s linearity, sensitivity, and detection performance across the tested range.

Pathogen	R-Squared	RMSE	Slope	Intercept	False Negative Concentration (CFU/mL)
*Campylobacter*	0.970	0.808	−2.119	29.62	None
*Salmonella*	0.856	1.853	−2.198	32.56	10^2^
*E. coli*	0.915	1.452	−2.323	34.51	10^2^
*Listeria* species	0.932	1.583	−2.856	37.69	10^2^
*Listeria monocytogenes*	0.808	4.118	−4.792	53.37	10^4^, 10^3^, 10^2^

**Figure A2:**
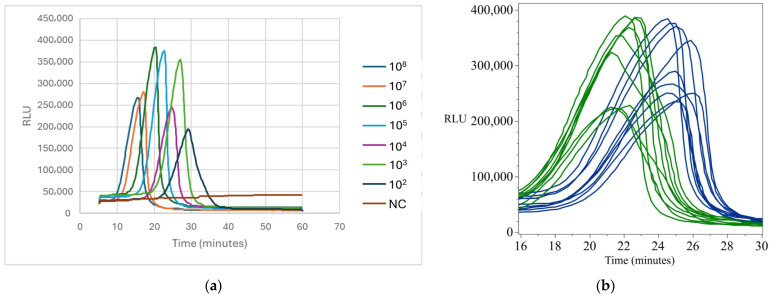
Standard and detection curves for *Campylobacter*. (**a**) shows the standard curve calibration with digitized data for different concentrations. (**b**) shows digitized detection plots *for Campylobacter* at 5 CFU/g (blue) and 25 CFU/g (green) inoculation levels.

**Figure A3:**
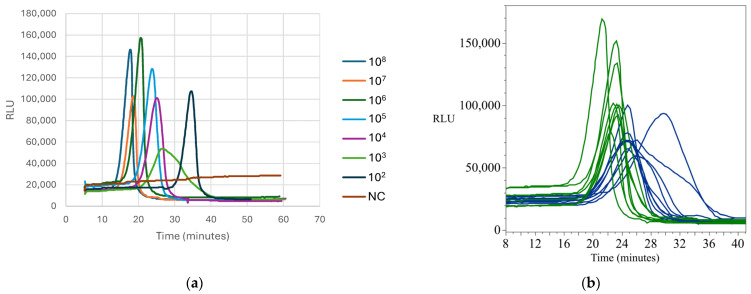
Standard and detection curves for *Salmonella*. (**a**) shows the standard curve calibration with digitized data for different concentrations. (**b**) shows digitized detection plots for *Salmonella* at 5 CFU/g (blue) and 25 CFU/g (green) inoculation levels.

**Figure A4:**
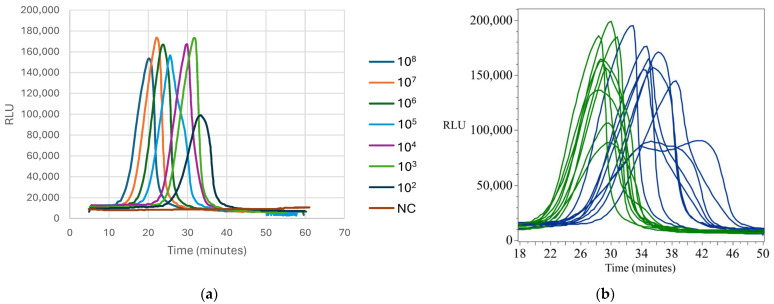
Standard and detection curves for *E. coli* O157. (**a**) shows the standard curve calibration with digitized data for different concentrations. (**b**) shows digitized detection plots for *E. coli* at 5 CFU/g (blue) and 25 CFU/g (green) inoculation levels.

**Figure A5:**
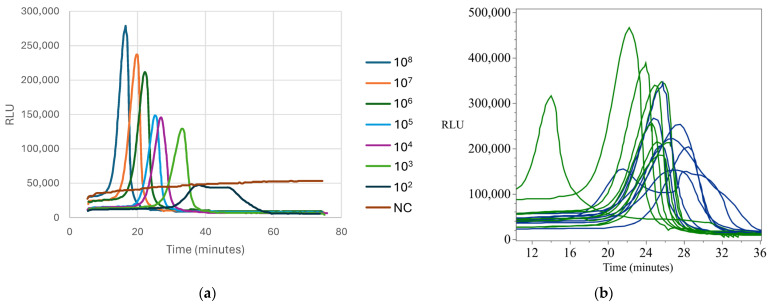
Standard and detection curves for *Listeria* Species. (**a**) shows the standard curve calibration with digitized data for different concentrations. (**b**) shows digitized detection plots for *Listeria* species at 5 CFU/g (blue) and 25 CFU/g (green) inoculation levels.

**Figure A6:**
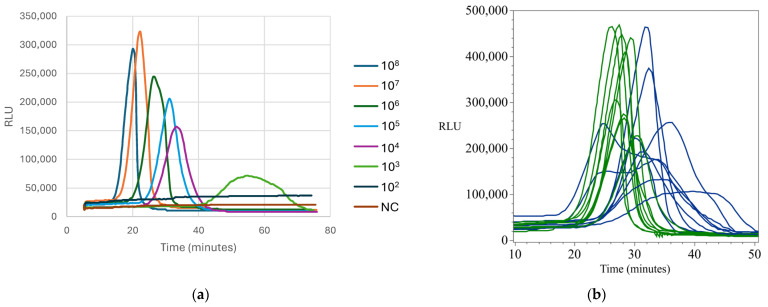
Standard and detection curves for *Listeria monocytogenes*. (**a**) shows the standard curve calibration with digitized data for different concentrations. (**b**) shows digitized detection plots for *Listeria monocytogenes* at 5 CFU/g (blue) and 25 CFU/g (green) inoculation levels.

## Data Availability

The original contributions presented in this study are included in the article. Further inquiries can be directed to the corresponding author.

## Appendix A

### Appendix A.1. Graph Digitizer

Plot digitization from the images provided in the Molecular Detection System (MDS) report was performed using PlotDigitizer Pro software. Images of MDS graphs were imported into the software, and axes were calibrated using known reference points from the graphs. Data points were digitized by tracing the curves with auto-detection algorithms, followed by manual refinements where needed. The extracted data were exported in a tabular format for further analysis to ensure accurate quantitative evaluation.

## Appendix B

This appendix provides visual representations of digitized calibration curves and detection plots for the tested pathogens. Panel (a) in each figure shows the digitized standard curves created during MDS calibration, while panel (b) illustrates the detection results from experiments involving inoculated chicken samples. Green curves correspond to samples inoculated at 25 CFU/g, and blue curves represent those inoculated at 5 CFU/g.
